# Investigation of bacterial nucleotide excision repair using single-molecule techniques

**DOI:** 10.1016/j.dnarep.2013.10.012

**Published:** 2014-01-25

**Authors:** Bennett Van Houten, Neil Kad

**Affiliations:** a Department of Pharmacology and Chemical Biology, University of Pittsburgh Cancer Institute, University of Pittsburgh, Pittsburgh, PA, USA; b School of Biological Sciences, University of Essex, Wivenhoe Park, Colchester CO4 3SQ, UK

**Keywords:** Bacterial nucleotide excision repair, UvrA, UvrB, UvrC, UvrD, Single molecule

## Abstract

Despite three decades of biochemical and structural analysis of the prokaryotic nucleotide excision repair (NER) system, many intriguing questions remain with regard to how the UvrA, UvrB, and UvrC proteins detect, verify and remove a wide range of DNA lesions. Single-molecule techniques have begun to allow more detailed understanding of the kinetics and action mechanism of this complex process. This article reviews how atomic force microscopy and fluorescence microscopy have captured new glimpses of how these proteins work together to mediate NER.

## 1. Introduction

### 1.1. Action mechanism of the bacterial UvrABC NER system: formation and disassembly of the machinery on DNA

**Fig. 1:**
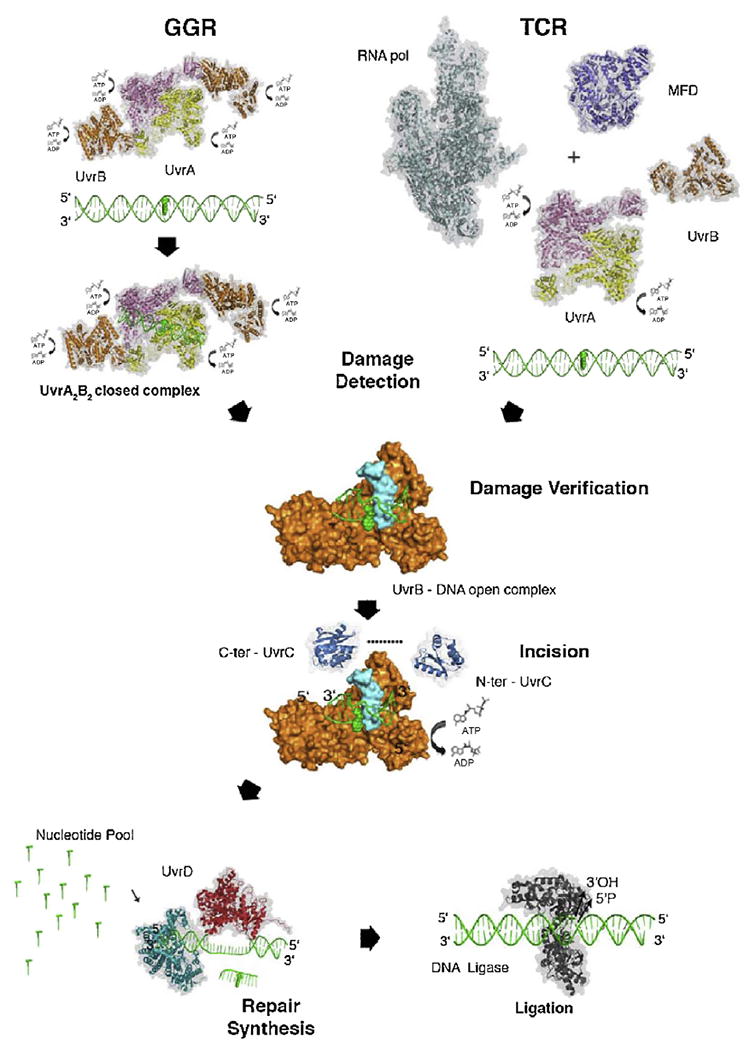
Prokaryotic nucleotide excision repair. Structural model of prokaryotic NER showing the key protein and steps in global genomic repair (GGR) and transcription coupled repair (TCR). TCR damage recognition is initiated by a stalled RNAP (PDB ID: 4LJZ) that recruits MFD (PDB ID: 2EYQ). MFD displaces RNAP and brings UvrA to the damaged site. In GGR, the UvrA_2_B_2_ complex (PDB ID: 3UWX) for the contact interface: 3FPN first searches for the distortion along the DNA caused by the lesion. Both pathways converge after the initial recognition steps. UvrA then transfers the damaged DNA to UvrB for damage verification. The dimeric UvrA protein (PDB ID: 2R6F) hydrolyzes both ATP and GTP. It also forms a complex with UvrB (PDB ID: 2FDC) and activates the ATPase activity of UvrB. During damage verification, the β-hairpin of UvrB (shown in *turquoise*) inserts between the two strands of DNA and forms a stable pre-incision complex, which is believed to activate UvrB’s ATPase. Binding and hydrolysis of ATP by UvrB is essential for recruitment of UvrC. The N-terminal endonuclease domain of UvrC (PDB ID: 1YCZ) initiates the cut 4–5 nucleotides 3′ to the damaged site followed by the 5′ cut by C-terminal endonuclease domain of UvrC (PDB ID: 2NRR) eight nucleotides away from the lesion. UvrD (PDB ID: 2IS1) unwinds the DNA and releases the oligonucleotide containing the lesion. Simultaneously, DNA polymerase I (PDB ID: 2HHQ) synthesizes the missing strand. Finally, DNA ligase I (PDB ID: 1DGS) seals the repair patch. All protein structures in this figure, with the exception of UvrB, are shown with a transparent surface and in ribbon presentation. UvrB is shown with its surface in orange for domains 1 to 3, and the β-hairpin is shown in cyan. C-ter, carboxy terminal; N-ter, amino terminal. From [6] with permission.

Prokaryotic nucleotide excision repair (NER) was reconstituted with six highly purified proteins in 1985 by the Grossman and Sancar laboratories [1,2]. Since that time a huge wealth of functional and structural information has accumulated on this system, reviewed in [3-6]. Bacterial NER is initiated in two ways: (i) during transcription when RNA polymerase encounters a progress blocking lesion in a process termed transcription-coupled repair (TCR); or (ii) when the UvrA_2_UvrB_2_ complex encounters a region of DNA which is distorted by the presence of a DNA lesion unconnected with transcription, this process is known as global genome repair (GGR). During the former, the TCR factor (Mfd) pushes RNA polymerase off from the lesion and recruits UvrA_2_ to the damaged site. Both GGR and TCR then proceed in a similar manner. In a step not well understood, the UvrA_2_ dimer passes the damaged region of DNA to UvrB, which uses a beta-hairpin to verify the damaged nucleotide on one of the two DNA strands [7,8]. Engagement of UvrB at the damage site facilitates UvrA_2_ dissociation and serves as a landing site for UvrC. UvrC is a dual nuclease which incises the damaged strand 3′ to the lesion using its N-terminal nuclease domain, and 5′ to the lesion using its C-terminal nuclease domain [9,10]. This post-incision UvrBC–DNA complex and an oligonucleotide containing the damage are dissociated by the dual action of UvrD and DNA polymerase I. DNA pol I fills in the excised region, and the repair patch is sealed by the action of DNA ligase [1,2] (see Fig. 1).

Despite almost three decades of research many fundamental questions remain unanswered regarding how the components of the prokaryotic NER machinery assemble at sites of damage [11]. These include: (i) how do DNA repair proteins, at levels of 100–1000 per bacterial cell, efficiently sort through a multi-million base pair genome for rare DNA lesions?; (ii) what are the dynamics of Mfd recruitment to a stalled RNA polymerase at a damaged site, and how/when are UvrA and UvrB subsequently recruited?; (iii) how and when is the lesion passed from UvrA to UvrB?; (iv) how is ATP binding is coupled to domain movement within UvrA and UvrB during damage engagement and verification?; (v) how does UvrD bind to the 5′ nick of the post-incision complex to allow dissociation of UvrC?; (vi) how is UvrB removed with the damaged oligonucleotides by the dual action of UvrD and DNA pol I?; and (vii) how is DNA ligase I recruited to the repair patch to seal the nick created by the action of DNA pol I? This review discusses how single-molecule techniques are being used to address these unanswered questions on the nature of the protein complexes and the kinetics of this dynamic process. Finally we discuss the outlook for the future of the field in which the entire process of prokaryotic nucleotide excision repair can be viewed one molecule at a time.

### 1.2. Toward a molecular movie of DNA damage recognition by UvrA and UvrB

The action mechanism of GGR and TCR has recently been reviewed [4,6,11] and the reader is encouraged to read those reviews for a more extensive description and citation list. However, briefly, UvrA is a member of the ATP-binding cassette superfamily of ATPases and binds DNA as a dimer, which stimulates its ATPase activity [12]. UvrA_2_ exhibits preferential binding to DNA lesions, but its overall lesion binding affinity appears independent of subsequent UvrB loading and incision [13-15]. UvrA_2_ makes extensive contacts along DNA with two charged residues providing strong binding energy for both non-damaged and damaged DNA, whereas the C-terminal zinc finger provides damage discrimination [16,17]. UvrA_2_ forms a complex with UvrB and the stoichiometry is believed to be 2UvrA:2UvrB [18,19]. As discussed below, this complex is believed to use both 3D-diffusion and 1 D-sliding to find DNA lesions [11,20]. See supplemental movie 1 for a molecular model showing the formation of the UvrA_2_UvrB_2_–DNA complex. In the absence of UvrA, UvrB is incapable of binding DNA through the action of its autoinhibitory domain 4 that also inhibits its ATPase activity [21]. However, once UvrA detects a lesion, UvrB, which shares a fold with superfamily 2 DEAD-box helicases, verifies the damaged site using its beta-hairpin that inserts directly into the double helix. Both UvrA and DNA damage stimulate the UvrB ATPase, which couples to the movement of UvrB’s helicase domain necessary for efficient binding to sites of damage and allowing UvrC to bind and trigger dual incisions [7]. UvrB is thought to verify lesions using several aromatic residues at the base of the beta-hairpin. In particular the completely conserved residue Tyr96 has been shown to be essential for damage discrimination and efficient UvrB–DNA complex formation [22,23]. See supplemental movie 2.

### 1.3. Nature of the damage: damage recognition is dynamic

One of the most remarkable features of NER is its ability to act on a wide variety of chemical and structurally dissimilar lesions. The question of how UvrA and UvrB can effectively process so many different types of substrates has been noted for over four decades [3,24,25]. Two key features of damage recognition are localized helical distortion and in most cases helical destabilization, both of which would facilitate opening of the DNA helix by UvrB’s beta-hairpin. In an attempt to understand the damage recognition process in more detail Geacintov and co-workers created benzo[a]pyrene diol epoxide dG adducts in two sequence contexts, CGC or TGT. Surprising the initial rates of incision by the UvrABC nuclease system were twofold slower for BPDE-dG in the context of CGC [26]. Molecular dynamic simulations of the BPDE-dG adducts within these two sequence contexts revealed that the amino groups of the two Gs (opposite the damaged dG) pinned the BPDE moiety into a conformation which showed very little movement. Whereas the BPDE-dG adduct in the context of the TGT sequence showed a much more dynamic structure undergoing large conformational changes over the course of the 10 ns simulation [25-27]. See supplemental movie 3. This remarkable finding indicates that damage verification by UvrB probes the dynamic nature of the DNA lesion in the DNA. It is believe that the human repair protein, XPC-HR23B uses a similar process [27].

### 1.4. New classes of damage added to the UvrABC substrate repertoire of lesions

**Fig. 2:**
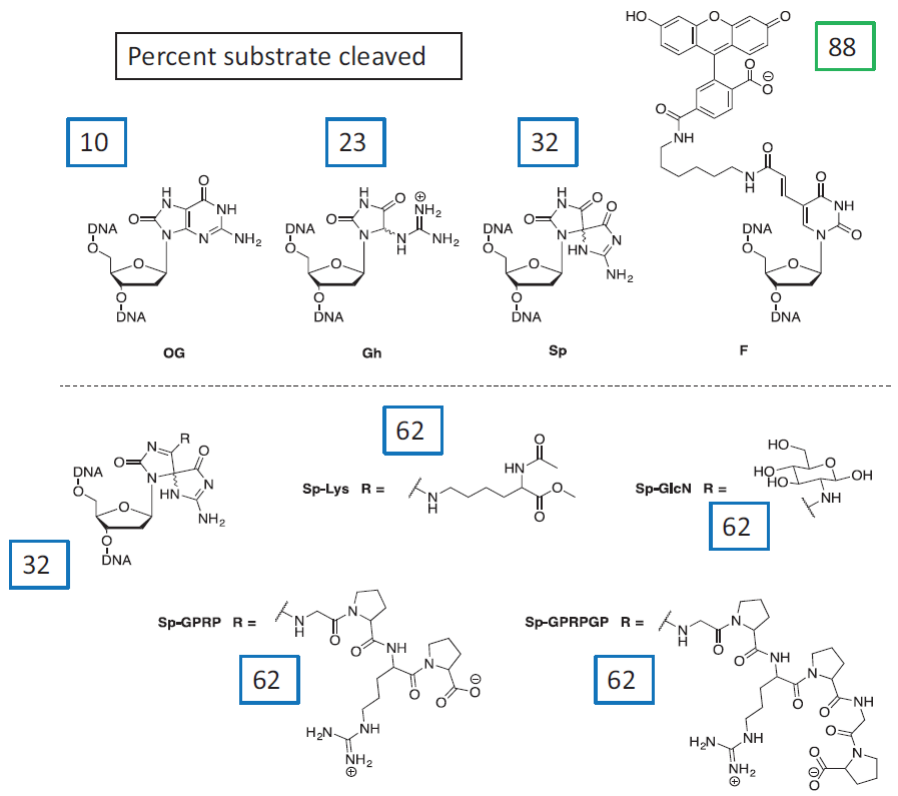
Oxidized bases recognized by the UvrABC system. While 8-oxo-dG (OG) is a poor substrate for the UvrABC system, further oxidation products of this adduct are good substrates [55]. These include, guanidinohydantoin (Gh) and the two diastereomers of spiroiminodihydantoin (Sp), and spiroiminiohydantoin-adducts: Sp-lys, Sp-GPRP, Sp-GlcN, and Sp-GPRPGP. The F, is a fluorescein-modified thymine which serves as a positive control. Numbers in boxes indicate the extent of incision of a DNA duplex containing these site-specific lesions. Adapted from [55] with permission.

**Fig. 3:**
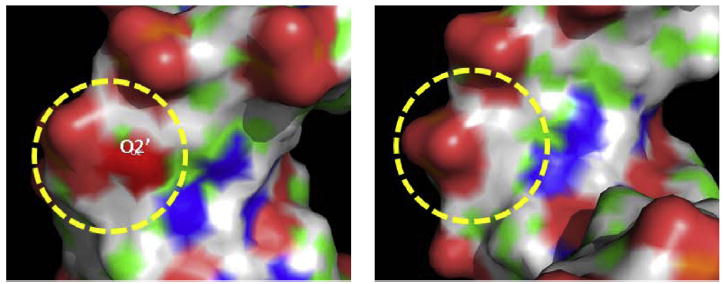
A single ribonucleotide is a robust substrate for the UvrABC system. Electrostatic surface for 2′-0H of the ribose moiety embedded in a DNA duplex. The red spot around the O2′ indicates the negative electrostatic potential. Prepared by Yuqin Cai, NYU See reference: Yuqin Cai, Nicholas E. Geacintov, Suse Broyde Ribonucleotides as nucleotide excision repair substrates. DNA Repair 13 (2014) 55–60.

This dynamic model of DNA damage recognition can also help explain the several new types of lesions that have been discovered to be good substrates for the UvrABC nuclease system. The growing repertoire of damage substrates are: protein–DNA cross-links [28-30], oxidized bases including: nitrosative stress [31], interstrand cross-links [32,33]; tandem base damages [34], and oxidized products of 8-oxodG (see Fig. 2). Finally, it has been recently shown that DNA polymerases occasionally insert ribonucleotides into DNA during DNA replication causing a highly mutagenic lesion [35,36]. A surprising observation is that while ribonucleases can actively remove ribonucleotides from DNA, NER provides an important back-up mechanism in prokaryotes for their removal [56] (see Fig. 3). How UvrB might be able to detect and process a ribonucleotide in the context of a DNA helix was nicely tested by molecular dynamic simulations using a UvrB–DNA complex and is reviewed by Yai and Broyde [57]. They suggest that the 2′-OH provides localized electro-negativity, stabilizing Tyr96 at the base of the beta-hairpin. This analysis also helps explain why DNA substrates containing a nick or a one base pair gap, which carry an extra-negative charge, are also recognized as a DNA lesion by the UvrABC system [12].

## 2. Dynamics of UvrA dimer on DNA

UvrA contains two ATP binding sites and three zinc fingers per monomer. The first crystal structure of UvrA from the thermophilic prokaryote *Bacillus stearothermophilus* (PDB entry 2R6F) from the Verdine group showed that UvrA formed an unusual dimer with the ATP signature sequence 1 binding to the second signature sequence to form a intramolecular ATP binding site [37]. The structure also revealed a potential cleft that could accommodate double-stranded DNA. Finally, the structure also revealed an independently folding domain that interacts with UvrB. This UvrA domain was later revealed to have extensive contacts with UvrB domain II [19]. Nowotny and co-workers recently solved the co-crystal structure of *Thermotoga maritima* UvrA_2_ bound to a DNA duplex containing a fluorescein-modified T (PDB entry 3PIH) on each strand, giving greater insight into damage recognition [17]. Comparing this new structure to the apo-UvrA_2_ structure revealed that the ATP binding domains were rigid, but several of the inserted domains were highly flexible and the position of the two UvrA monomers relative to each other moved to accommodate the DNA double-helix within the cleft as predicted by the Verdine structure. The C-terminal zinc fingers which are necessary for damage recognition were found to swing out of the way of the DNA to allow contacts with the cleft and key positively charged residues. The DNA was found to be unwound by about 20° and bent by about 15°. This cleft appeared to be perfectly suited to allow one-dimensional sliding, but as discussed below UvrA once bound to DNA is static.

In order to study how UvrA can sort through a vast genome of millions of base pairs to find rare lesions we used DNA tightropes (described in chapter 1 of this volume) to study how single molecules of UvrA and UvrB scan DNA in search of lesions [20]. Briefly, lambda DNA (48.5 kbp) was suspended between 5 μm beads coated with poly-l-lysine which had been immobilized to a microscope coverslip. The resulting DNA tightropes were stained with the DNA intercalating YOYO-1 dye and observed using oblique angle fluorescence microscopy. In order to study the UvrA, UvrB and UvrC proteins in real time as they interrogated DNA for damage we developed several strategies to conjugate quantum dots (Qdots) to these repair proteins. For UvrA we attached a biotin ligase recognition sequence (GLNDIFEAQ**K**IEWHEGGG, Avi-Tag™) to the C-terminal end of the protein. Co-expression of the biotin ligase during overproduction of UvrA results in >90% conjugation of UvrA with a single biotin on the lysine residue of this sequence. Streptavidin-coated Qdots can then be used in excess to assure that only one UvrA dimer binds to a single Qdot. Less than 5% of UvrA molecules when bound to lambda DNA showed any diffusion on DNA. Most showed transient binding with an average life time of 7 s. Thus UvrA searches for DNA lesions by rapid three-dimensional diffusion and short lived sampling of the DNA. Due to the high concentration of DNA on our tightrope platform we were able to observe UvrA jumping from one double helix to another (see supplementary movie 4) with an average jump distance of 1.2 μm. These data indicated that UvrA, even under SOS induced levels of ~200 copies per cell, could not adequately search the entire bacterial genome to allow efficient repair. In the next section we will see that UvrB provides a new function to UvrA to allow efficient searching for DNA damage.

## 3. Role of UvrB in dynamic DNA damage recognition

UvrB is essential for damage verification and serves as a platform for UvrC binding and subsequent nuclease activity. Using atomic force microscopy Wyman and Goosen were able to show that DNA is wrapped around UvrB and that two UvrB molecules within the UvrAB complex allows inspection of the each strand for the precise site of the damaged nucleotide [38,39]. Using capillary electrophoresis coupled with laser-induced fluorescence polarization, which combines a mobility shift assay with conformational analysis, Weinfield and co-workers demonstrated that DNA wrapping around UvrB, was mediated by UvrA [40]. There has been some confusion in the literature about the stoichiometry of UvrB in the absence of DNA or UvrA. Gel filtration chromatography and velocity sedimentation experiments indicate UvrB is a monomer in solution, whereas atomic force microscopy suggested that UvrB can form dimers potential through the highly flexible coiled-coiled domain 4 which also acts as an inhibitory domain [38,41]. Using gel mobility shift assay, Moolenaar and Goosen were able to show that at high concentrations of UvrB, dimers form on DNA [42]. NMR analysis of methyl-^13^C methionine labeled UvrB indicated that while domain 4 can interact with UvrB to form chemical shifts suggesting dimerization, two independent monomers of UvrB could not form dimers in solution even at high concentrations of protein necessary for NMR experiments. Most recently, however, dimerization of UvrB has been recently observed by Barrett and co-workers who obtained a new crystal structure of UvrB in conjunction with ssDNA and the non-hydrolyzable ATP analog, AMPPCP [43]. They also used chemical cross-linking electron paramagnetic resonance spectroscopy to confirm that presence of UvrB dimers in solution.

**Fig. 4:**
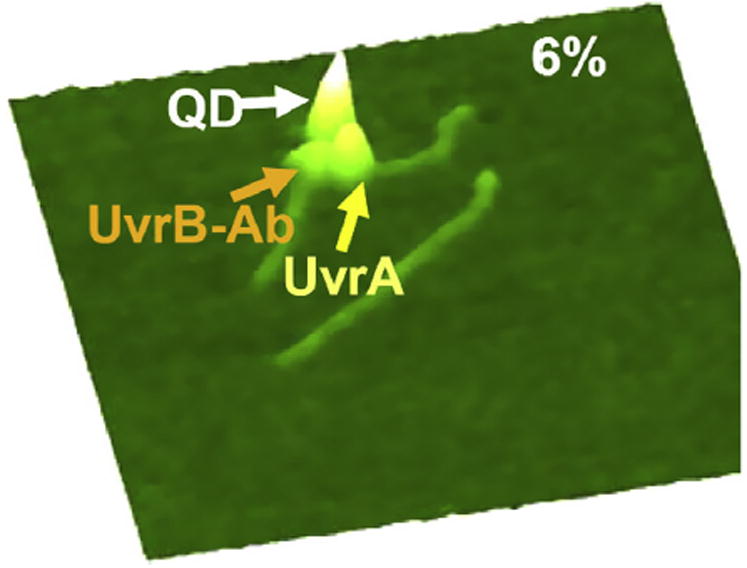
UvrAB complex with UvrB-Qdot. A hemagglutinin (HA) epitope tag (YPYD-VPDYA) was engineered on to the N-terminus of UvrB to which was conjugated a mouse monoclonal antibody. A goat antimouse coated Qdot was bound to the UvrB-HA-Ab to make an “antibody” sandwich. This UvrB-HA-Ab-Ab-Qdot complex was mixed with UvrA and a 517 bp fragment prepared by PCR and containing a nick 40% from one end. This AFM image shows the transient complex of UvrA loading UvrB-HA-Ab-Ab-Qdot at the site of a nick, only 6% of the total complexes found on DNA had both UvrB and UvrA bound at the site of the nick. Adapted from [44] with permission.

In order to observe UvrB interactions with UvrA on DNA, we devised a second strategy for conjugating Qdots to repair proteins [44] as shown in Fig. 4 – UvrAB complex with UvrB-Qdot. In this approach we engineered a hemagglutinin (HA) epitope tag (YPYDVPDYA) on to the N-terminus of UvrB to which was conjugated to a mouse monoclonal antibody. A goat anti-mouse coated Qdot was bound to the UvrB-HA-Ab to make an “antibody” sandwich. In this way we could use two differently colored Qdots to follow UvrB in solution [20]. We could not observe any colocalization of two differently labeled UvrB molecules, however when UvrA was added colocalization of UvrB molecules was evident. Goosen and co-workers did similar experiments with fluorescently labeled UvrB and found similar results [45]. These data would suggest that at low concentrations UvrB does not form dimers, but can readily form a UvrA_2_UvrB_2_ complex.

**Fig. 5:**
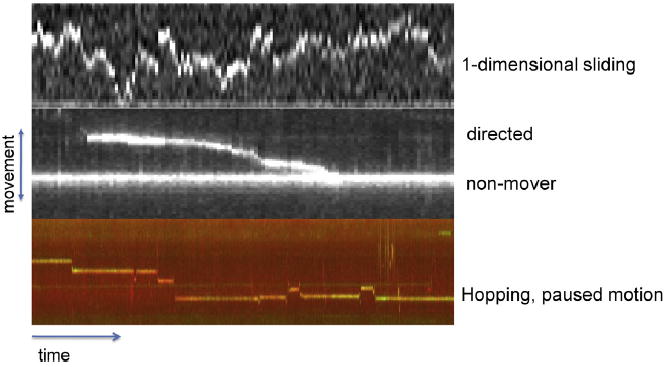
Nature of UvrAB movement on nondamaged DNA. UvrA shows no mobility once bound to DNA. Addition of UvrB to UvrA resulted in longer lived complexes with an average lifetime of 40 s. About 17% of the complexes showed motility on DNA exhibiting a range of motions including one-dimensional diffusion, directed motion that was ATP-dependent, and paused motion on the same DNA molecule following by rapid excursions to a new position on the DNA, hopping.

Using Qdot-labeled UvrB we saw no evidence that UvrB could interact with DNA in the absence of UvrA. When UvrA and UvrB were observed binding to DNA about 17% of the molecules were found to be highly mobile on the DNA displaying several modes of movement on the DNA as shown in the kymographs displayed in Fig. 5 [11,20] (see supplemental movie 5). The UvrAB complexes were longer lived than UvrA alone, ~40 s versus 7 s. The principle type of motion of UvrAB molecules on DNA was a one-dimensional random walk with a relatively slow diffusion constant of 3.5 × 10^−3^ μm^2^ s^−1^. This slow diffusion indicated a significant diffusional energy barrier of 3.9 k_B_T. These data would suggest that domains of the UvrAB complex are involved in probing the DNA helix for distorted bases during the sliding motion. Using UvrA conjugated to green Qdots and UvrB to red Qdots we were able to see transient binding of both proteins at specific sites on lambda DNA followed by subsequent UvrA departure leaving UvrB on DNA (see supplemental movie 6: UvrA loading of UvrB).

## 4. Observing UvrBC on DNA

**Fig. 6:**
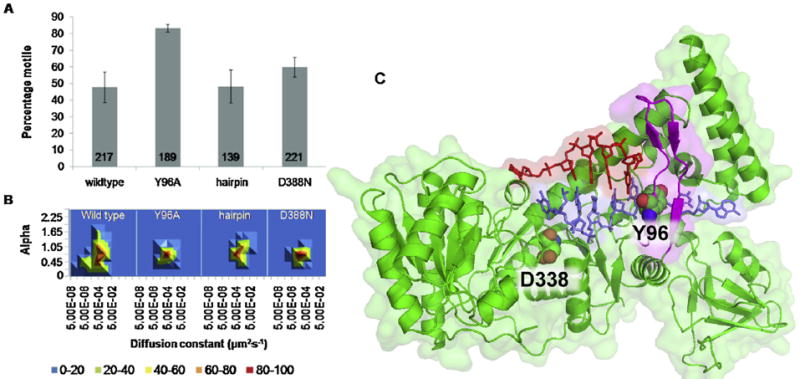
Role of UvrB motifs in UvrBC movement. To conjugate UvrC to Qdots, the biotin ligase recognition sequence GLNDIFEAQKIEWHEGGG (AviTag™) was fused to the C-terminus of *Bacillus caldotenax* UvrC. At 50 mM KCl UvrC alone showed avid DNA binding, but no DNA sliding. *Panel A*. Addition of WT, or one of several mutant UvrB: Y96A, beta-hairpin deletion (Δhairpin) or D338N, resulted in DNA sliding. *Panel B*. 3D density plots of the diffusion constant versus the alpha factor for each UvrBC mutant complex. The coloring is a percentage scale relative to the maximum bin size. *Panel C*. UvrB–DNA co-crystal (PDB, 2FDC) Cartoon model with transparent surface of UvrB (green); non-damaged DNA strand (red); damage containing DNA strand (blue); beta-hairpin (magenta). Y96 and D338 show as space fill. Adapted from [46] with permission.

Our long term goal is to watch the entire process of prokaryotic NER at the single molecule level and to this end we have conjugated Qdots to UvrC using the avitag strategy of biotin ligase and streptavidin-Qdots. We found that UvrC binds avidly to double-stranded DNA and at 50 mM KCl only 15% of the molecules showed mobility on the DNA [46], and their mean attached lifetime of was ~30 s. Surprisingly when UvrB-Qdots conjugates were added to UvrC-Qdot conjugates in solution we found complexes of UvrB and UvrC bind to DNA with a much greater degree of motility (~35–60% – see supplemental movie 7). However, the attached lifetimes of the complexes these remained around 30 s. Under no circumstances could we observe UvrB binding to UvrC molecules that were already attached to the DNA. This suggests that UvrC once bound to DNA sterically hinders the UvrC coiled-coiled domain from interacting with the C-terminal coiled-coiled domain of UvrB to form a UvrBC complex. Analysis of the motion of the UvrBC complexes on DNA showed striking heterogeneity with a range of diffusion constants over several orders of magnitude (not uncommon in determinations of diffusion constants). Further analysis of the dynamics of the UvrBC complex on DNA indicated two populations of molecules some of which showed highly diffusive motion and other molecules having a sub-diffusive stop–start motion. Raising the salt concentration from 50 mM to a more physiological 150 mM KCl decreases the lifetimes of the UvrC and UvrBC complexes on DNA and also produces faster diffusion. An increase in the diffusive exponent toward one was also observed, indicating a more random diffusive process without a stop–start motion. In order to assess whether UvrB as part of the UvrBC complex was making significant contact with DNA, three UvrB mutations were evaluated: a beta-hairpin deletion, a Y96A substitution and an ATPase dead mutant, D338N. As shown in Fig. 6 the UvrBC complexes diffused faster without the beta-hairpin or with the Y96A mutation. These data strongly suggest that UvrB is making contact through the beta-hairpin and some of the start-stop sub-diffusive behavior could be due to UvrB’s dragging this domain through the DNA.

## 5. UvrD mechanics of motion

UvrD is a superfamily 1 helicase member and moves in a 3′ → 5′ direction to displace DNA in an ATP dependent reaction [47]. This multi-tasking helicase plays key roles in NER and mismatch repair. During NER UvrD is believed to displace UvrC and the Adapted from [20] with permission. oligonucleotide containing damage [48,49]. However, the reaction stoichiometry is not known and UvrD in the absence of other factors is poorly processive from a nick. Interestingly MutL has been shown to increase UvrD’s ability to displace a DNA strand from a nick [50]. With regard to NER it is not clear whether the post-incision complex of UvrB facilitates binding of UvrD to the 5′ nick site or how UvrD is recruited to the post-incision complex; either through 3D-diffusion or 1D sliding. UvrD has been shown to interact with UvrB and the UvrAB complex has been shown to stimulate UvrD’s helicase activity [18,51]. In a set of amazing crystal structures, Yang and co-workers were able to acquire snap-shots of UvrD during its catalytic cycle which they spliced together to create a molecular movie [52]. ATP binding was found to induce a large conformational change in the protein which causes 1 bp opening of the duplex. Subsequent ATP hydrolysis allows translocation of UvrD in the 3′ → 5′ direction.

Single molecule analysis of UvrD was first achieved using magnetic tweezers by the Croquette group in which double stranded DNA was attached to a magnetic bead on one end and to a glass surface on the other end [53]. The DNA is then stretched by applying force to the magnetic bead. UvrD unwinds from a nick and causes a change in the bead position which can be accurately monitored in all three spatial dimensions. These studies suggested a step-size of about 6 bp per catalytic cycle and that unwinding occurs at ~41 bp/s. They also made the surprising finding that UvrD undergoes strand switching and causing bursts of re-zipping of unzipped stretches. These important studies were limited by the inability to observe UvrD in action as it translocated. In an exciting recent study the Ha group combined optical tweezers with single-molecule fluorophore tracking to show that UvrD as a monomer is a highly processive translocase, able to move on single strand DNA at a rate of 193 nt/s [54] with an average processivity of 1260 bases. Furthermore they showed that single monomers of UvrD were stalled at a single-strand–double-strand junction, and a second molecule of UvrD is required to bind to allow unwinding of the double-stranded DNA and establish a new slower rate of unwinding with an average of 70 bp/s, but showed remarkable heterogeneity in the rates of unwinding.

## 6. Outlook and unresolved questions

Single molecule approaches have begun to allow direct visualization of Uvr protein–DNA intermediates during the process of NER. But at the same time single molecule approaches have thrown open a whole new series of important questions, including: (i) what is the nature of the heterogeneity in the movement of UvrAB on DNA?; (ii) how does damage move from UvrA to UvrB?; (iii) what is the role of UvrBC on DNA?; (iv) how does UvrC find the UvrB–DNA complex, and what is the rate of the dual incision reaction?; and finally (v) what are the kinetics and the precise role of UvrD and DNA pol I in the turnover of the UvrBC post incision complex? Once DNA with defined lesions can be readily strung-up in our tightrope system we will be able to begin to dissect these important steps of NER and fully reconstitute this process one molecule at a time using a rainbow of Qdot conjugated proteins.

## Supplementary Material

Movie 1

Movie 2

Movie 3

Movie 4

Movie 5

Movie 6

Movie 7
