# Supplementary figures and images for: Investigation of bacterial nucleotide excision repair using single-molecule techniques
Source: DNA Repair (Amst). Author manuscript; Available in PMC 2016 Oct 6. (PMC5053424; doi:10.1016/j.dnarep.2013.10.012)

## Slide 1
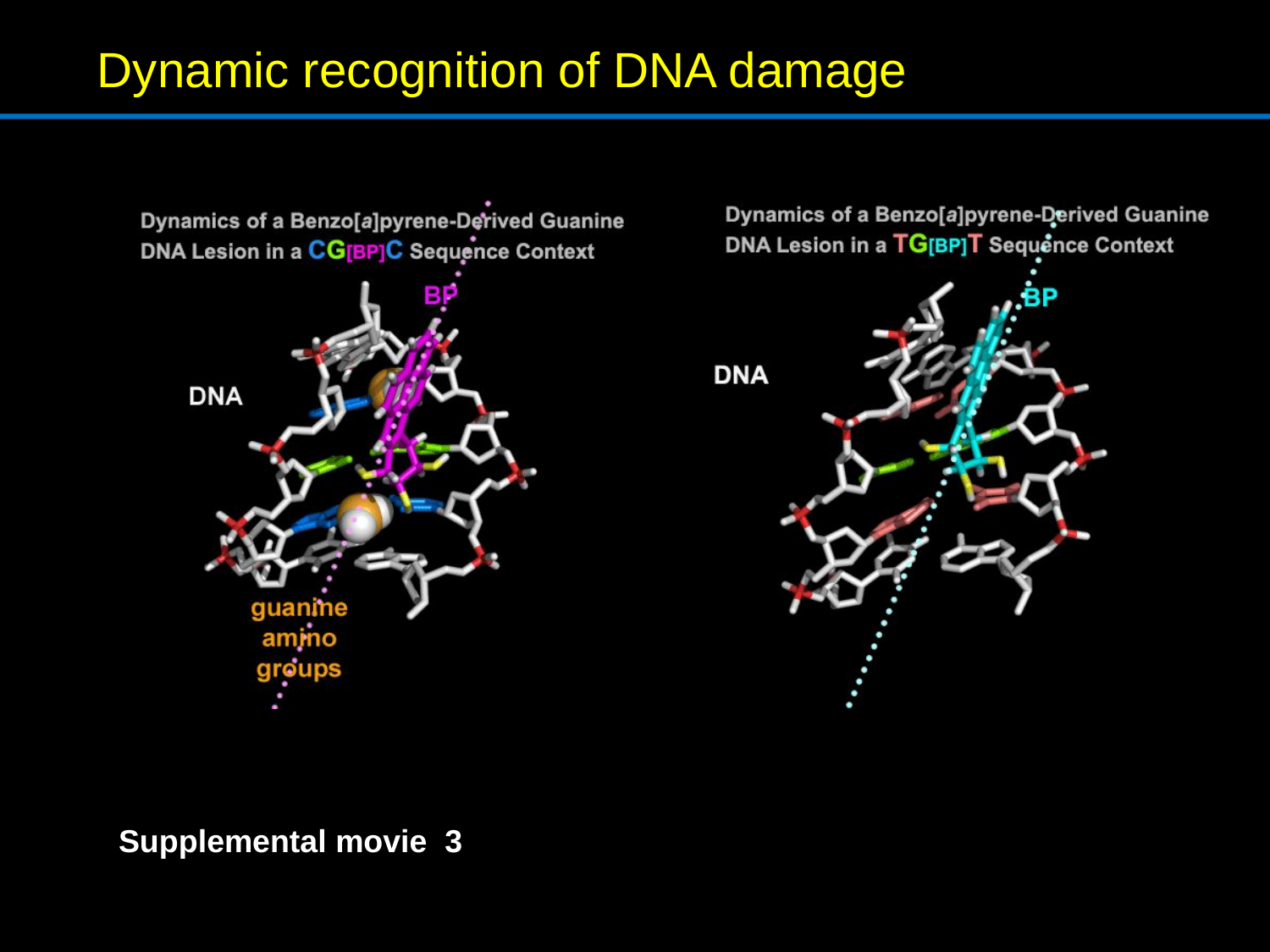

Dynamic recognition of DNA damage
Supplemental movie 3

Supplement: Movie 3 [file NIHMS818047-supplement-Movie_3.pptx]

## Slide 1
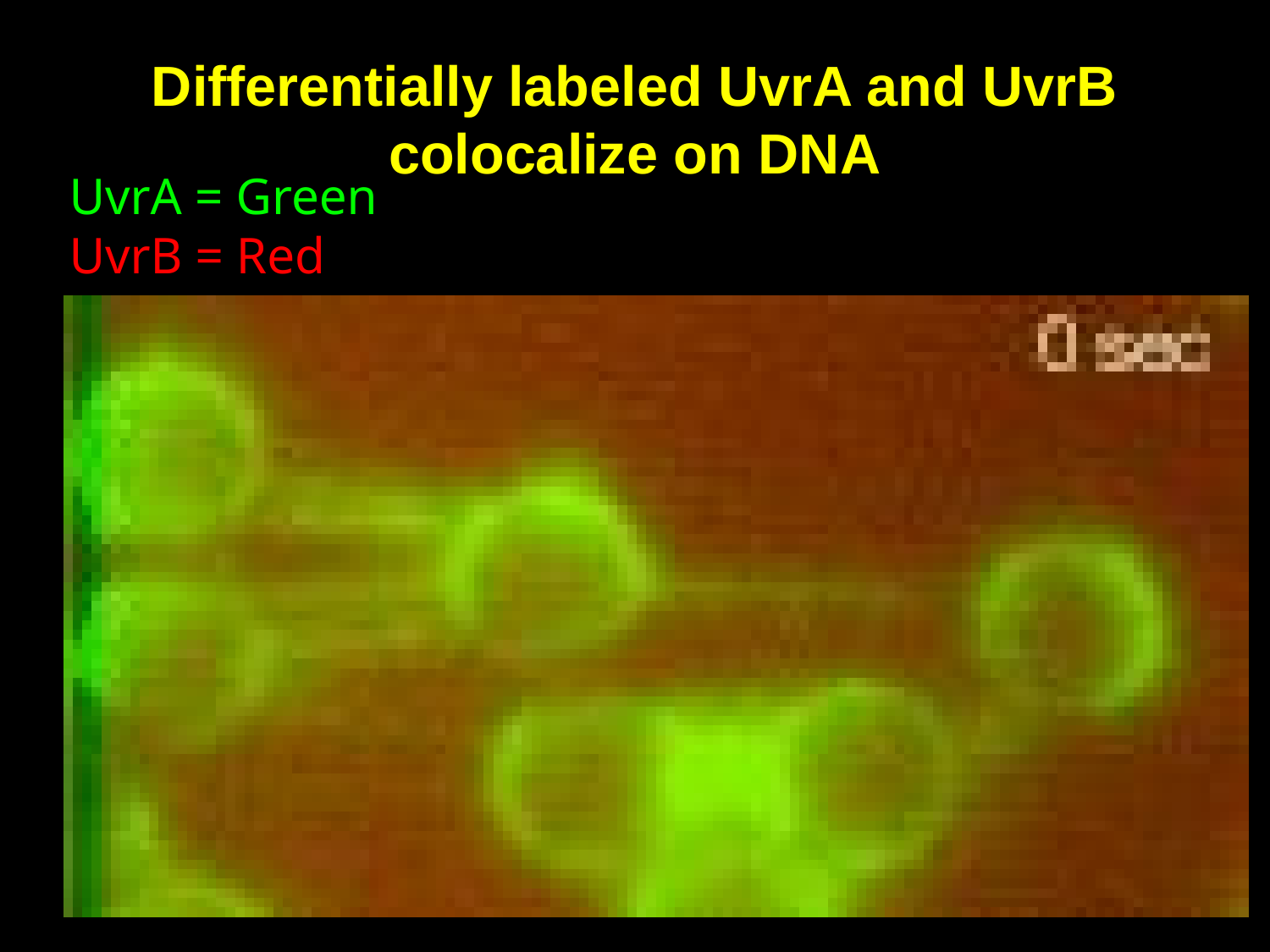

# Differentially labeled UvrA and UvrB colocalize on DNA
UvrA = Green
UvrB = Red

Supplement: Movie 6 [file NIHMS818047-supplement-Movie_6.pptx]
